# *In Silico* Genotyping of Escherichia coli Isolates for Extraintestinal Virulence Genes by Use of Whole-Genome Sequencing Data

**DOI:** 10.1128/JCM.01269-20

**Published:** 2020-09-22

**Authors:** Anna Maria Malberg Tetzschner, James R. Johnson, Brian D. Johnston, Ole Lund, Flemming Scheutz

**Affiliations:** a Research Group for Genomic Epidemiology, Technical University of Denmark, Kongens Lyngby, Denmark; b Minneapolis Veterans Affairs Health Care System, Minneapolis, Minnesota, USA; c University of Minnesota, Minneapolis, Minnesota, USA; d The International Centre for Reference and Research on Escherichia and Klebsiella, Department of Bacteria, Parasites and Fungi, Statens Serum Institut, Copenhagen, Denmark; National Institute of Allergy and Infectious Diseases

**Keywords:** ExPEC, *in silico*, virulence typing, whole-genome sequencing

## Abstract

Extraintestinal pathogenic Escherichia coli (ExPEC) is the leading cause in humans of urinary tract infection and bacteremia. The previously published web tool VirulenceFinder (http://cge.cbs.dtu.dk/services/VirulenceFinder/) uses whole-genome sequencing (WGS) data for *in silico* characterization of E. coli isolates and enables researchers and clinical health personnel to quickly extract and interpret virulence-relevant information from WGS data. In this study, 38 ExPEC-associated virulence genes were added to the existing E. coli VirulenceFinder database. In total, 14,441 alleles were downloaded. A total of 1,890 distinct alleles were added to the database after removal of redundant sequences and analysis of the remaining alleles for open reading frames (ORFs). The database now contains 139 genes—of which 44 are related to ExPEC—and 2,826 corresponding alleles. Construction of the database included validation against 27 primer pairs from previous studies, a search for serotype-specific P fimbriae *papA* alleles, and a BLASTn confirmation of seven genes (*etsC*, *iucC*, *kpsE*, *neuC*, *sitA*, *tcpC*, and *terC*) not covered by the primers. The augmented database was evaluated using (i) a panel of nine control strains and (ii) 288 human-source E. coli strains classified by PCR as ExPEC and non-ExPEC. We observed very high concordance (average, 93.4%) between PCR and WGS findings, but WGS identified more alleles. In conclusion, the addition of 38 ExPEC-associated genes and the associated alleles to the E. coli VirulenceFinder database allows for a more complete characterization of E. coli isolates based on WGS data, which has become increasingly important considering the plasticity of the E. coli genome.

## Abstract (precis)

Extraintestinal pathogenic Escherichia coli (ExPEC) is the leading cause in humans of urinary tract infection and bacteremia. The previously published web tool VirulenceFinder (http://cge.cbs.dtu.dk/services/VirulenceFinder/) uses whole-genome sequencing (WGS) data for *in silico* characterization of E. coli isolates and enables researchers and clinical health personnel to quickly extract and interpret virulence-relevant information from WGS data.

## INTRODUCTION

Extraintestinal pathogenic Escherichia coli (ExPEC)—those E. coli strains with an enhanced ability to cause infections outside of the intestine—is by far the leading cause of urinary tract infection (UTI) (hence the label uropathogenic E. coli [UPEC]) and bacteremia (1). E. coli isolates are typically classified operationally as ExPEC based on their presumed intrinsic virulence potential as inferred from the presence/absence of specific putative or proven virulence genes irrespective of their immediate source of isolation. Thus, a molecular typing tool is needed for determination of whether an anonymous isolate is likely ExPEC or non-ExPEC. In contrast with ExPEC, the E. coli strains that cause diarrhea are referred to collectively as diarrheagenic E. coli (DEC) or intestinal pathogenic E. coli (IPEC). Additionally, in contrast with both ExPEC and DEC, nonpathogenic commensal E. coli strains colonize the human intestine without causing disease and may even be beneficial to the host by harvesting energy, protecting against other pathogens, or regulating host immunity. There is, however, a thin line between the definition of virulence and fitness factors in ExPEC and commensals. It has been suggested that ExPEC virulence might be a by-product of the commensal lifestyle (2).

Pathogenic and nonpathogenic E. coli strains differ with respect to their repertoire of virulence traits. Molecular epidemiological analyses have shown that ExPEC is quite distinct overall from commensal and DEC (or IPEC) in terms of pathogenic potential, ecology, evolution, reservoirs, transmission, pathways, host-pathogen interactions, and virulence mechanisms (3). The interaction between the bacteria and the host is a complex and multifactorial process involving adhesins, siderophores, toxins, protectins (including lipopolysaccharides [LPS] and capsules), invasins, and various other putative virulence and/or colonization factors.

To allow presumptive classification of E. coli isolates as to extraintestinal virulence potential, two main operational definitions have been derived by comparing limited sets of virulence genes with epidemiological and infection model data. According to these definitions, isolates are classified as (i) ExPEC_JJ_ if positive for two or more of *papAH* and/or *papC* (P fimbriae), *sfa-focDE* (S and F1C fimbriae), *afa-draBC* (Dr-binding adhesins), *iutA* (aerobactin siderophore system), and *kpsM* II (group 2 capsules) (4), and as (ii) UPEC_HM_ if positive for two or more of *chuA* (heme uptake), *fyuA* (yersiniabactin siderophore system), *vat* (vacuolating toxin), and *yfcV* (adhesin) (5). These definitions tend to identify highly similar, albeit nonidentical, groups of isolates.

Whole-genome sequencing (WGS) has provided a better understanding of the core and accessory genomes of pathogenic and commensal E. coli isolates and has allowed for the development of PCR primers and probes for a large number of virulence genes. The number of described PCR targets for putative and proven extraintestinal virulence genes is up to 57 (6), which makes PCR-based virulence profiling of ExPEC isolates challenging. WGS is increasingly being used to characterize E. coli isolates, including conventional seven-locus multilocus sequence typing (MLST) (http://enterobase.warwick.ac.uk/species/index/ecoli) and core genome MLST (cgMLST) (http://pubmlst.org/databases/), *in silico* serotype prediction (7), major phylogenetic group (8), and virulence gene detection (9).

The Center for Genomic Epidemiology (CGE) provides the publicly available, user-friendly web tool VirulenceFinder, which enables detection of virulence genes in WGS data from E. coli (9). The program detects virulence genes by either performing a BLAST search against assembled genome data or aligning raw reads with *k*-mer alignment (KMA) (10) against a FASTA database containing the virulence genes. VirulenceFinder was established to meet the need for quick virulence gene detection and typing to the allele level using WGS data.

The first version of VirulenceFinder included the most important gene markers for the four most important human DEC pathotypes as follows: enteropathogenic E. coli (EPEC) (including attaching and effacing E. coli [AEEC]), enterotoxigenic E. coli (ETEC), enteroinvasive E. coli (EIEC), and Shiga toxin-producing E. coli (STEC), which is also referred to as Vero cytotoxin-producing E. coli (VTEC). It also included markers for multiple animal-associated ETEC fimbriae, 11 serine protease autotransporters of *Enterobacteriaceae* (SPATE) genes, and an arbitrary selection of colicins. In 2015 and 2016, 28 genes of enteroaggregative E. coli (EAEC), a fifth human DEC pathotype, including the five AAF fimbriae genes, were added, and all *prfB* variants were removed because they had been entered by mistake. Also, in 2015, 144 *stx* holotoxin alleles were added to the database. With these changes, the E. coli virulence gene database contained 936 alleles representing 101 genes, plus 144 *stx* holotoxin alleles. Of these, only six genes (*cnf1*, *hlyE*, *ireA*, *iroN*, *iss*, and *sfaS*) were ExPEC associated.

Due to increasing reports from users that more ExPEC-associated genes were needed to allow a more complete characterization of their WGS data, we began to work on adding more ExPEC genes to the tool. The present report describes the first major addition of alleles to the E. coli component of the curated VirulenceFinder database since its initial development in 2014 (9) and the subsequent addition in 2016 of virulence genes associated with EAEC.

Here, we identified 38 ExPEC-associated genes (1,890 alleles) that we considered relevant to add to the E. coli VirulenceFinder database for rapid and easy *in silico* determination of molecular ExPEC status and detailed characterization of presumptive ExPEC isolates. The expanded database, containing 139 genes and 2,826 alleles, was evaluated and validated by comparing PCR results with *in silico* findings from 9 control strains and 288 molecularly defined ExPEC and non-ExPEC strains (6, 11).

## MATERIALS AND METHODS

### Study design and isolates.

To supplement VirulenceFinder's existing E. coli virulence gene database as of study onset (101 genes, 936 alleles), a supplemental ExPEC FASTA database containing a selection of diverse ExPEC-associated genes was constructed. Genes were identified as candidates for inclusion based on the genes used in the two main established operational definitions for ExPEC and UPEC (4, 5) and recommendations from expert colleagues (authors J. R. Johnson and B. D. Johnston as well as Erick Denamur [INSERM, Universités Paris Diderot et Paris Nord, France] and David M. Gordon [Ecology and Evolution, Research School of Biology, the Australian National University, Acton, Australia]). As a proof of concept, the database was validated first by comparing PCR virulence genotyping results obtained in previous studies for nine control strains (6) with the virulence genes predicted here *in silico* for the same nine strains by using the revised VirulenceFinder to analyze the WGS data of these strain. A second evaluation was done by comparing (previous and new) PCR virulence genotyping results for 288 clinical and fecal strains of human origin that had been classified previously as ExPEC_JJ_ versus non-ExPEC_JJ_ (11) with the virulence genes predicted here by applying the revised VirulenceFinder to the WGS data of these strains. Finally, using WGS-based pathotype classifications as derived using the revised VirulenceFinder, the ExPEC_JJ_/non-ExPEC_JJ_ status of these 288 strains was compared with their UPEC_HM_/non-UPEC_HM_ status.

### Control stains for validation.

The initial proof of concept analysis used nine strains—hereafter termed control strains—that had previously been classified by multiplex PCR as representing ExPEC_JJ_. They were included for validation of 18 singleton genes and genes representing two operons (*foc-sfa* and *afa-dra-daa*) in the VirulenceFinder ExPEC database (6). Of the nine control strains, five (BioProject accession numbers PRJNA169903, PRJNA475142, PRJNA479435, PRJNA475142, PRJNA16235) had publicly available genomes that, for this study, were collected from NCBI, whereas two (11A and 31A) underwent WGS within this study. One of the nine control strains (L31) ultimately was excluded for reasons described in Text S1 in the supplemental material. Additionally, strain JJ055 (positive for *fimH* and *ompT*) was replaced by K-12 strain MG1655 (GenBank accession number U00096.3) as a non-ExPEC negative control.

### Evaluation strain sequences and PCR results.

In the second evaluation, 288 strains of human origin (179 ExPEC_JJ_ strains and 109 non-ExPEC_JJ_ strains) (11) with publicly available genomes (NCBI) (12) and virulence gene PCR results (11) were included. The PCR results included presence/absence of the *sfa-foc* and *afa* operons and 14 individual genes, as determined by the use of 22 primer pairs, including five primer pairs for variants of *kpsM* and *kpsMT*. Multiplex PCR genotyping of these evaluation strains was performed as described previously (11).

### Whole-genome sequencing.

Control strains 11A and 31A were sequenced using an Illumina NextSeq (Illumina, San Diego, CA, USA). The sequences were *de novo* assembled and their MLST determined (Text S1 in the supplemental material).

### ExPEC gene database.

An ExPEC FASTA database with selected ExPEC-associated genes was constructed. Gene alleles were added to the already established E. coli VirulenceFinder database at CGE web tools. Gene names were changed according to decisions described in the results section.

### Building the ExPEC database.

A preliminary FASTA database was constructed by searching the NCBI GenBank nucleotide collection (https://www.ncbi.nlm.nih.gov/) for all entries containing the selected genes in E. coli and that had been deposited before October 2018. The following search string was used: ((Escherichia coli[Organism]) AND “genename”[Gene Name]) AND (“0001/01/01"[Publication Date] : “2018/01/10"[Publication Date]). Only complete genes were collected. All unique gene alleles were added to the ExPEC database. Multiple alignment and identity matrices of database gene variants were performed by using MUSCLE (13) with default parameters. For those ExPEC genes with previously described PCR primers (6, 14, 15), the corresponding sequences were sought in the database by using the publicly available tool MyDBFinder (version 1.2) (https://cge.cbs.dtu.dk/services/MyDbFinder/), with thresholds of 90% identity and a minimum length of 60%.

A total of 9,589 out of 11,170 gene alleles were removed from the candidate ExPEC database. After removal of redundant gene alleles, this included gene alleles for which primers were available but for which (i) one or both primers could not be located in the gene sequence and (ii) the alleles shared less than 60% identity with other gene alleles in which one or both primer sequences were located (Table 1). The threshold of 60% was chosen to maximize the database validity. The *yfcV* variants with less than 85% identity to sequences containing the PCR primer sequences were excluded from the ExPEC database to avoid detection of the cryptic *yfcV* gene described in E. coli K-12 (5) and possibly other nonpathogenic or commensal E. coli strains. The primers used for PCR identification of *cia* and *cib* (14) bind outside the gene sequence and could not be used for validation of the *cia* and *cib* alleles. Accordingly, the published *cia* and *cib* alleles (16) were used to curate the database, and alleles with an identity of >90% were included in the database. No primer sequences were available for the following seven genes: *etsC*, *iucC*, *kpsE*, *neuC*, *sitA*, *tcpC*, and *terC*. These genes were validated by BLASTx against the nonredundant protein sequences database (nr) by using BLAST (https://blast.ncbi.nlm.nih.gov/Blast.cgi) and a threshold of more than 60% identity within gene alleles. Table 1 lists the ExPEC genes added to VirulenceFinder.

**TABLE 1:** Gene content of the ExPEC database downloaded from NCBI and added to the VirulenceFinder database

Gene	Description	No. from NCBI	No. in database	Identity (%)
*afaA*	Transcriptional regulator	14	4	68
*afaB*^*a*^	Periplasmic chaperone	12	3	69
*afaC*	Outer membrane usher protein	13	11	69
*afaD*	Afimbrial adhesion	62	37	46
*afaE*	Adhesin protein	42	30	43
*cea*	Colicin E1	132	23	63
*chuA*	Outer membrane hemin receptor	423	79	75
*cia*	Colicin Ia	259	37	58
*cib*	Colicin Ib	24	6	98
*clbB*	Hybrid nonribosomal peptide/polyketide megasynthase	270	77	97
*cvaC*	Microcin C	166	10	90
*etsC*	Putative type I secretion outer membrane protein	169	18	47
*focC*	S fimbrial/F1C minor subunit	710	2^*b*^	99
*focC*/*sfaE*	S fimbrial/F1C minor subunit		2^*b*^	
*focG*	F1C adhesion	9	2	99.8
*focI*	S fimbrial/F1C minor subunit	5	1	
*fyuA*	Siderophore receptor	465	98	97
*hlyF*	Hemolysin F	287	21	65
*hra*	Heat-resistant agglutinin	132	11	89
*ibeA*	Invasin of brain endothelial cells	369	66	97
*irp2*	High-molecular-weight protein 2 nonribosomal peptide synthetase	1,033	346	97
*iucC*	Aerobactin synthetase	335	47	95
*iutA*	Ferric aerobactin receptor	350	71	70
*kpsE*	Capsule polysaccharide export inner membrane protein	54	21	49
*kpsM*	Polysialic acid transport protein	94	82	47
*mcbA*	Bacteriocin microcin B17	949	2	98
*neuC*	Polysialic acid capsule biosynthesis protein	961	68	45
*ompT*	Outer membrane protease (protein protease 7)	3,564	314	66
*papA*^*c*^	Major pilin subunit	116	42	54^*d*^
*papC*	Outer membrane usher P fimbriae	786	40	50
*sfaD*	S fimbrial/F1C minor subunit	18	10	98
*sfaE*	S fimbrial/F1C minor subunit	3	1^*b*^	98
*sfaS*	Sialic acid-binding adhesion	54	1	99.8^*e*^
*sitA*	Iron transport protein	369	56	91
*tcpC*	Tir domain-containing protein	24	3	99
*terC*	Tellurium ion resistance protein	126	25	52
*traT*	Outer membrane protein complement resistance	1,386	200	40
*usp*	Uropathogenic-specific protein	19	6	91
*yfcV*	Fimbrial protein	768	14	87.8
Total		14,441	1,890	

a The three *afaB* genes are more than 67% identical to the five already included *nfaE* alleles in the original VirulenceFinder database.

b Two *sfaE* and *focC* alleles were 100% identical and are called *focC/sfaE* in the database.

c Including one *fteA* (F10), two *feiA* (F8), two *fsiA* (F16) one *ffiA* (F15), and one *ffoA* (F14).

d Identity was below 60% for two new *papA* alleles (see Text S1 in the supplemental material).

e Identity to the already included allele in the original VirulenceFinder database.

Dr adhesins were located using E. coli strain HM358 (GenBank accession number JN688153.1), for which the entire Dr locus had been sequenced, and each of the genes (*afaABCDE* and *draP*, complete coding DNA sequence [cds]) were used for a BLASTn search at NCBI. Subsequently, gene designations *afaE*, *afaE*-8, and *afaE*-VIII were sought in the NCBI nucleotide database. The sequences identified for each hit were extracted and divided into each locus for the entire *afa*, *dra*, and *daa* operons. The Dr adhesin genes were then curated to share an *afaABCDE* nomenclature, except for the original five *nfaE* alleles, which were kept as in the original E. coli VirulenceFinder database (see Results).

### Validation and evaluation of the ExPEC database for *in silico* typing of ExPEC strains.

The final ExPEC virulence gene database was added to the existing E. coli VirulenceFinder database (https://bitbucket.org/genomicepidemiology/virulencefinder_db/src/master/) (9). WGS sequences were uploaded to VirulenceFinder as either assembled genomes (control strains) or raw reads (evaluation strains) using a threshold of 80% identity and a minimum length of 60%. Strains were classified as ExPEC_JJ_ if positive for ≥2 of the following: *papAH* and/or *papC* (P fimbriae), *sfa-focDE* (S and F1C fimbriae), *afa-draBC* (Dr-binding adhesins), *iutA* (aerobactin siderophore system), and *kpsM* II (group 2 capsules) (6). Strains were considered positive for *afa-draBC* if a combination of *afaB* or *nfaE* and also *afaC* was identified and for the *sfa-focDE* operon by WGS if a combination of *focC* or *sfaE* and also *focI* or *sfaD* was identified. Strains were classified as UPEC_HM_ if positive for two or more of the following: *chuA* (heme uptake), *fyuA* (yersiniabactin siderophore system), *vat* (vacuolating toxin), and *yfcV* (adhesin) (5).

### Data availability.

Whole-genome sequences from strains 31A and 11A are available at the European Nucleotide Archive (accession number PRJEB38689) with accession numbers ERS4600802 (strain 31A) and ERS4600803 (strain 11A). The new E. coli VirulenceFinder database (virulence_ecoli.fsa) and the associated notes file (notes.txt) can be downloaded from https://bitbucket.org/genomicepidemiology/virulencefinder_db/src/master/.

## RESULTS

To augment the existing E. coli VirulenceFinder database (101 genes, 936 alleles), a supplemental ExPEC FASTA database consisting of 38 ExPEC-associated genes (including 1,898 alleles and 1 updated *sfaS* allele) was constructed. For this, 14,441 alleles of the 38 genes of interest were downloaded from NCBI. Removal of redundant alleles and analysis of the remaining alleles for open reading frames (ORFs) left a total of 1,890 distinct alleles (Table 1), which will be added to the E. coli VirulenceFinder database as of this paper's date of acceptance. The newly augmented E. coli VirulenceFinder database will contain 2,842 alleles of 139 putative or confirmed virulence genes, of which 75 are DEC-associated, 44 are ExPEC-associated, and 20 are found in almost all E. coli strains, irrespective of pathotype.

### The ExPEC database.

Construction of the ExPEC database was based on the 27 published primer pairs (for *afaE8*, *afa*, *kpsMT* III, *yfcV*, *ibeA*, *fyuA*, *clbB*, *sfa-focDE*, *iutA*, *hra*, *ompT*, *kii*, *papC*, *kpsM*-K5, *cvaC*, *focG*, *traT*, *sfaS*, *kpsM*-K1, *kpsII*, *usp*, *chuA*, *kpsM*-K15, *hlyF*, *irp2*, *papA*, and *papA* F type-specific) used to detect ExPEC virulence genes by PCR in the control strains (6) plus seven genes (*etsC*, *iucC*, *kpsE*, *neuC*, *sitA*, *tcpC*, and *terC*) without published primers. The search string used for *papA* did not return the expected allele results for the differently-named, serotype-specific *papA* variants *feiA* (F8), *fteA* (F10), *ffiA* (F15), *fsiA* (F16) (15), and *ffoA* (F14), which instead were added after a search for these alleles in NCBI GenBank (Text S1 in the supplemental material). From these searches, the total number of gene alleles downloaded from NCBI was reduced from 14,441 candidate alleles to 1,890 curated alleles (Table 1). The following sections list the number of *sitA*, *sfa-focDE*, and *afa-dra-daa* operons and *kpsM*, *cia-cib*, and *hra* genes (see Text S1 in the supplemental material for details of the analyses). Text S1 in the supplemental material also describes analysis and inclusion/exclusion of alleles for the remaining ExPEC genes downloaded from NCBI.

### *sitA*.

Fifty-six unique *sitA* alleles were added to the database.

### *sfa-focDE* operon.

The published *sfa-focDE* primers were designed to bind to *sfaD* and *sfaE* in the *sfa* (S fimbriae) operon (17), which in the *foc* (F1C fimbriae) operon correspond with *focI* and *focC*, respectively (18). Here, *sfaD*, *sfaE*, *focI*, and *focC* were downloaded to represent the *sfa-focDE* primers for the consensus region shared between the *sfa* and *foc* operons.

Of 80 unique putative *focC* alleles downloaded from NCBI, 76 were not included in the final ExPEC database. A total of 10 unique *sfaD* alleles and 1 *focI* allele were downloaded from NCBI. In summary, 2 *focC*, 2 *focI*, 10 *sfaD*, 1 *sfaE*, and 2 *focC/sfaE* alleles were added to the ExPEC database.

The original E. coli VirulenceFinder database contained one *sfaS* allele. The present study identified one additional unique *sfaS* allele (identity, 99.8%) and two additional unique *focG* alleles (identity, 99.8%). The three new alleles were added to the ExPEC database. In principle, finding a combination of *sfaE/focC* and *sfaD-focI* in a queried sequence when using VirulenceFinder should indicate the presence of the *sfa* and/or *foc* operon(s), but this awaits assessment by future users.

### *afa-dra-daa* and *aggB*.

Four unique *afaA* alleles, originally designated as *afaA*-3-*draA*-3, *daaA*, *afaA*-1, and *afaA*-8, were all designated as *afaA* in the ExPEC database. Because three of the *afaB* alleles from NCBI were 100% identical to three *nfaE* alleles already present in the E. coli VirulenceFinder database, only 3 new *afaB* alleles (two *afaB*-1 alleles, one *afaB*-8 allele) were added to the ExPEC database. Eleven *afaC* alleles were added to the ExPEC database, including three unique alleles each for *afaC*-1, *afaC-draC*, and *afaC*-8 and two for *afaC*-3. A total of 37 unique *afaD* alleles were added to the ExPEC database, including alleles classified as Agg3B, Agg4/HdaB, and/or *afaD*, and the corresponding allele labels were changed to *afaD*.

Thirty unique *afaE-draE-daa* alleles were added to the ExPEC database. These included 14 unique *afaE3*-*draE* alleles, 9 Dr adhesin (*afa-dra*) alleles, and 1 *daaE* allele (F1845) as well as 1 *afaE1*, 1 *afaE2*, 1 *afaE5*, and 3 *afaE8* alleles. Text S1 in the supplemental material provides more details regarding the included *afa-dra-daa* and *aggB* alleles.

### *kpsM*.

In the search for *kpsM* alleles, preference was given to sequences for which information was available regarding the serotype, including the K capsule antigen and/or the original K antigen reference strain number (see appendix 3 in reference 19). The nucleotide sequences of the *kpsM* alleles clustered together in three distinct groups as follows: group 2 contained 68 *kpsM* alleles, group 3 contained 7 alleles, and *kpsM*-15 contained 3 alleles. Four additional unique alleles included two for the group 3 capsule K19 and one each for the group 2 capsules K94 and K97. Text S1 in the supplemental material provides more details regarding the *kpsM* alleles.

### *cia-cib*.

Six *cib* alleles (98.0 to 99.9% identity to one another) and 38 *cia* alleles (58.9 to 99.9% identity to one another) were added to the ExPEC database. Text S1 in the supplemental material provides more details regarding the *cia* and *cib* alleles.

### *hra*.

The study's initial search string identified only one *hra* allele, which originated from porcine ExPEC strain PCN033 (20). Accordingly, an additional BLASTn search was performed using the *hra* sequence from PCN033; this identified 131 additional *hra* alleles with <72% identity to the *hra* allele in PCN033 and 95.5% identity to the *hra* allele found in EAEC strain 60A (21). Eleven of the 131 candidate *hra* alleles were added to the ExPEC database (see Text S1 in the supplemental material for details).

### Validation of ExPEC genes identified by VirulenceFinder in the control strains.

After the above adjustments, the final ExPEC database was added to the E. coli VirulenceFinder database, and both the newly added and the preexisting ExPEC alleles were used first to search and validate available WGS data and PCR results from the nine control strains. These included 18 singleton genes (*afaE8*, *chuA*, *clbB*, *cvaC*, *fyuA*, *hlyF*, *hra*, *ibeA*, *irp2*, *iutA*, *kpsM*, *ompT*, *papA*, *papC*, *sfaS*, *traT*, *usp*, *yfcV*) and two operons (*sfa-foc* and *afa-dra-daa*) (6) (control strain L31 was excluded as explained in Text S1 in the supplemental material).

Overall, with the nine control strains, concordance was high between typing results obtained *in vitro* by PCR versus *in silico* by VirulenceFinder. Specifically, VirulenceFinder was able to assign 17 (of 18) genes and both operons from the ExPEC database in concordance with the PCR results, i.e., yielded concordance for *afaE8*, *afa-dra*, *chu*A, *clbB*, *cvaC*, *focG*, *fyuA*, *hlyF*, *hra*, *ibeA*, *iutA*, *sfa-focDE*, *sfaS*, *traT*, *usp*, and *yfcV*. Table 2 shows a by-strain comparison between PCR and WGS for detection of the genes originally found by PCR and the additional genes found by WGS (Table S1 in the supplemental material lists the complete revised VirulenceFinder typing results for the control strains.)

**TABLE 2:** Comparison of the typing results for the nine control strains by PCR versus WGS typing by strain, serotype and ExPEC_JJ_/UPEC_HM_ status

Isolate no., serotype, ExPEC_jj_/UPEC_HM_ status	Concordance, WGS and PCR	PCR only	WGS only^*a*^	WGS additional genes^*b*^
11A,^*c*^ O9a:H9, non-ExPEC_JJ_/non-UPEC_HM_	*hra*, *traT*	*ompT*		*cia*, *terC*, *fanA* (F5), *f17A*, *f17G*, *fim41a*
2H16,^*d*^ O25:K2:H2, ExPEC_JJ_/non-UPEC_HM_	*afa-draBC*,^*e*^ *fyuA*, *iutA*, *ompT*, *papC*, *traT*, *kpsM* II		*papA*, *clbB*	*afaA*, *afaD*, *afaE*, *clbB*, *irp2*, *iucC*, *kpsE*, *sitA*, *terC*, *fsiA*-F16
2H25, O18ac:K1:H7, ExPEC_jj_/UPEC_HM_	*chuA*, *clbB*, *fyuA*, *hra*, *ibeA*, *kpsM*-K1, *ompT*, *papAH*,^*f*^ *papC*, *sfa-focDE*,^*g*^ *sfaS*, *traT*, *usp*, *yfcV*			*irp2*, *kpsE*, *neuC*, *terC*, *fteA*-F10
31A, ONT:H9, non-ExPEC_JJ_/non-UPEC_HM_	*fyuA*, *hra*, *iutA*, *ompT*, *papC*, *traT*		*papA*	*iucC*, *sitA*, *terC*, *f17A*, *f17G*, *fim41a*
536, O6:K15:H31, ExPEC_jj_/UPEC_HM_	*kpsM*-K15, *chuA*, *clbB*, *fyuA*, *hra*, *ompT*, *papAH*,^*f*^ *papC*, *sfa-focDE*,^*g*^ *sfaS*, *usp*, *yfcV*			*irp2*, *kpsE*, *sitA*, *tcpC*, *terC*, *papA*-F536
J96, O4:K-:H5; F1C:F13, ExPEC_jj_/UPEC_HM_	*chuA*, *clbB*, *focG*, *fyuA*, *hra*, *kpsM* III, *papAH*,^*f*^ *papC*, *sfa-focDE*,^*g*^ *traT*, *usp*, *yfcV*		*ompT*	*cea*, *irp2*, *kpsE*, *sitA*, *tcpC*, *terC*, *papA*-F13
K-12,^*h*^ Orough:H48, Non-ExPEC_JJ_/non-UPEC_HM_	*ompT*			*terC*
PM9, O9:K34:H-, ExPEC_JJ_ by WGS only/non-UPEC_HM_	*afaE8*, *cvaC*, *fyuA, hlyF*, *hra*, *iutA*, *ompT*, *traT*		*afaB*, *afaC*, *papC*	*afaA*, *afaD*, *afaE*, *etsC*, *hlyF*, *irp2*, *iucC*, *sitA*, *terC*, *f17A*, *f17G*
V27, O2:K5:H1, ExPEC_jj_/UPEC_HM_	*kpsM*-K5, *chuA*, *clbB*, *ficG*, *fyuA*, *iutA*, *ompT*, *papA*,^*c*^ *papC*, *sfa-focDE*,^*g*^ *usp*, *yfcV*			*cea*, *irp2*, *iucC*, *kpsE*, *sitA*, *tcpC*, *terC*, *fteA*-F10, *papA*-F14

a These genes were sought by PCR.

b These genes were not sought by PCR and only identified by use of the added ExPEC genes and alleles to the original VirulenceFinder database. See Table S1 for a complete list of genes.

c This strain, isolated from calf diarrhea, is a typical ETEC strain and also positive for *sta1* (heat-stabile enterotoxin ST-Ia).

d This strain, isolated from urine, is a typical EAEC strain that is positive for *aggR*, *aap*, *aar*, *aatA*, *pic*, *sat*, *sepA*, and *astA*.

e Strains were considered *afa-draBC*-positive by WGS if *afaB* or *nfaE* and *afaC* was present in the strain.

f Strains were considered positive for *papAH* by WGS if *papA* was identified.

g Strains were considered positive for the *sfa-focDE* operon by WGS if a combination of *focC* or *sfaE* and *focI* or *sfaD* was identified.

h The strain K-12 was not subject to the PCR protocol used to identify ExPEC genes in the control strains. PCR findings were based on NCBI annotation and compared with the WGS findings in this study.

Regarding discrepancies between PCR and WGS results, in only one instance did VirulenceFinder fail to identify a gene previously identified by PCR (*ompT* in strain 11A). By contrast, VirulenceFinder identified six ExPEC genes that PCR had not previously identified, including the following (strain number): *afaB* (PM9), *afaC* (PM9), *clbB* (2H16), *ompT* (J96), *papA* (31A and 2H16), and *papC* (PM9). Possible explanations for these discrepancies are addressed here by gene. (i) In strain PM9, for unclear reasons, PCR detected only *afaE8* where WGS found also *afaA*, *afaB*, *afaC*, *afaD*, and *afaE*, indicating the presence of the full *afa-dra* operon. (ii) In strain 2H16, the analyzed sequence contained both the forward and reverse *clbB* primer sequences, leaving unexplained the negative PCR result. (iii) In strain J96, the sequence contained only the forward *ompT* primer sequence, explaining the negative PCR result. (iv) In strains 31A and 2H16, *papA* contains only the forward *papA* primer sequence; the reverse primer sequence is within *papH* (6), which is not included in the ExPEC database. Per WGS data, *papH* in both strains (31A and 2H16) differs by one nucleotide from the *papH* primer sequence, potentially explaining the negative *papAH* PCR result despite a positive *papA* result in VirulenceFinder. (v) In strain PM9, the *papC* allele identified by VirulenceFinder lacked the *papC* PCR primer sequences, explaining the strain's negative PCR result. (vi) Strain K-12 was not subject to the PCR protocol used to identify ExPEC genes in the control strains (6), so PCR findings were imputed based on NCBI annotation and compared with the present WGS findings. A cryptic *yfcV* gene is annotated in K-12 (5), but the *yfcV* primer sequences (6) are not present in this cryptic sequence and also were not detected by WGS. For all control strains, ExPEC_JJ_/UPEC_HM_ classifications were concordant across detection methods (PCR versus WGS) except with strain PM9, which by PCR was ExPEC_JJ_ negative but by WGS contained *papC* and, therefore, qualified as ExPEC_JJ_. Additionally, regarding DEC pathotype classifications, strain 2H16 qualified as typical enteroaggregative E. coli (EAEC), as it was positive for *aggR*, *aap*, *aar*, *aatA*, *pic*, *sat*, *sepA*, and *astA*, and strain 11A qualified as a typical ETEC strain, as it was positive for *sta1* (heat-stabile enterotoxin ST-Ia).

### Comparison of PCR versus VirulenceFinder for ExPEC gene detection in the 288 evaluation strains.

As a second validation approach, for the 288 evaluation strains, the virulence genotyping results obtained previously by BLAST analysis (for *yfcV* and *chuA*) and PCR (all other virulence genes) (11) were compared to the results obtained here using the curated, revised VirulenceFinder (including the ExPECFinder database). Table 3 shows the concordance of PCR typing and the expanded VirulenceFinder detection for the 179 ExPEC and 109 non-ExPEC evaluation strain sequences (Table S2 in the supplemental material shows genes identified by VirulenceFinder). Overall, 5,934 (93.7%; per strain median, 95.5%) of 6,333 total positive and negative gene reactions were concordant by PCR and VirulenceFinder; only 399 (6.3%) were discordant. For seven randomly selected strains with one or more PCR-WGS typing discrepancy, PCR was repeated to assess the basis for the discrepancy. Repeat PCR improved concordance for *clbB*, *fyuA*, *hra*, *kpsM*-K1, *ompT*, *papC*, *sfa-focDE*, *sfaS*, *traT*, and *usp*. Including the repeat PCR results, PCR and WGS identified the same genes in 107 strains (median, 7 genes/strain), disagreed for one or two genes in 130 strains (of 9 [median] total genes/strain), and disagreed for ≥3 genes in 47 strains (of [median] 11 total genes/strain). Inclusion of these repeat PCR results resolved 135 (33.8%) of the 399 initial PCR-WGS discrepancies, leaving only 264 (4.2% of 6,333) (see Table S3 in the supplemental material).

**TABLE 3:** Concordance for the 288 evaluation strains by PCR versus WGS typing

Virulence gene	No. of virulence genes identified by PCR, WGS, or both^*a*^		
	Concordance, WGS and PCR (%)	PCR only	WGS only
*papAH*	249 (86.5)	5	34
*papC*	265 (92.3)	2	20
*sfa-focDE*	277 (96.2)	3	8
*sfaS*	281 (97.6)	2	5
*focG*	276 (95.8)	2	10
*afa-draBC*	275 (95.5)	7	6
*afaE8*	287 (99.7)	1	0
*hra*	271 (94.1)	5	12
*hlyF*	282 (97.9)	3	3
*fyuA*	275 (95.5)	3	10
*iutA*	266 (92.4)	1	21
*kpsM* II	275 (95.5)	6	7
*kpsMT* III	288 (100.0)	0	0
K1 *kpsM*	271 (94.1)	15	2
K5 *kfiC*	257 (88.9)	1	30
K15 *kpsM*	288 (100.0)	0	0
*cvaC*	281 (97.6)	0	7
*usp*	271 (94.1)	8	9
*traT*	254 (88.2)	7	27
*ibeA*	278 (96.5)	3	7
*ompT*	252 (87.5)	3	33
*clbB*	273 (94.8)	2	13
Average	272 (94.6)		

a Concordance (%) is calculated based on agreement between PCR and WGS findings. If both methods identify or do not identify a gene, it is classified as concordance (see Table S3 in the supplemental material). PCR results are those after PCR was redone for 7 strains.

### Discrepancies between PCR- and WGS-based typing.

Among the 288 evaluation strains, we found that 152 (58%) of the 264 residual PCR-WGS typing discrepancies involved WGS-identified genes that, paradoxically, contained both primer sequences. In descending order of frequency, these genes and operons (number of strains) were *iutA* (21), K5 *kfiC* (19), *traT* (16), *clbB* (13), *ompT* (12), *hra* (12), *papC* (11), *focG* (10), *fyuA* (10), *sfa-focDE* (8), *ibeA* (7), *sfaS* (5), *hlyF* (2), K1 *kpsM* (2), *usp* (2), *kpsM* II (1), and *afa-draBC* (1) (see Table S3). We have no explanation for these discrepancies.

By contrast, a possible explanation was apparent for the 112 (42% of 264) remaining PCR-WGS discrepancies (see Table S3). (i) For 68 (26% of 264) PCR-WGS discrepancies, the implicated target gene contained only one primer sequence. Of these, fully half (*n* = 34) involved *papA*; the identified sequences contained the forward *papA* PCR primer sequence, but because *papH* was not searched for using KMA, presence of the *papH* reverse primer sequence was not assessed. Thus, the negative PCR result for these strains could indicate either that *papH* is not always adjacent to *papA* or that the reverse *papH* primer fails to detect certain *papH* variants. The 34 remaining “single-primer-only” discrepancies involved (number of strains) *ompT* (13), *usp* (7), *cvaC* (5), *kpsM* II (4), K5 *kfiC* (3), *hlyF* (1), and *traT* (1). (ii) For 44 (17% of 264) PCR-WGS discrepancies, the implicated target gene contained neither primer sequence. These involved (number of strains) *traT* (10), *papC* (9), K5 *kfiC* (8), *ompT* (8), *afa-draBC* (5), *kpsM* II (2), and *cvaC* (2) (see Table S3).

For *kpsM*, PCR and WGS were concordant for 275 (95%) strains, whereas for *kpsM*-K15 and *kpsM* III, they were 100% concordant. For other *kpsM* variants, most PCR-WGS discrepancies involved disagreement regarding only the specific K type. For example, WGS identified *kpsM*-K5 in eight strains that per previous PCR results contained *kpsM*-K1 (for a more detailed description of the *kpsM* findings, see Text S1 in the supplemental material).

Discrepancies also occurred with identification of the *afa-draBC* operon. The operon was accepted as being present if VirulenceFinder identified both *afaB* (or *nfaE*) and *afaC* because these are the PCR primer targets. Inexplicably, VirulenceFinder identified only *afaA* and *afaC* (without *afaB*) in six *afa-draBC* PCR-positive strains (236_PUTI, 940_FVEC, H2_BUTI, PM3_BUTI, 408_PUTI, and 77_Pyelo). Likewise, VirulenceFinder identified *afaA* and *afaC* (without *afaB*) in two *afa-draBC* PCR-negative strains (142_PUTI and 89_PYELO). Finally, VirulenceFinder identified both *afaB* and *afaC* in five *afa-draBC* PCR-negative strains (1187_VA1000, 1291_VA1000, H27, U6, and 1631_FVEC_Fecal). The alleles identified by VirulenceFinder lacked the corresponding PCR primer sequences, explaining their nondetection by PCR.

### Identifying ExPEC_JJ_ strains among the 288 evaluation strains.

For additional validation, the 288 evaluation strains were assessed for ExPEC_JJ_ status by both PCR and VirulenceFinder using the established molecular definition of ExPEC_JJ_ (4). After repeat PCR (see Text S1 in the supplemental material; Table 3), 185 (64.2%) of the strains qualified as ExPEC_JJ_ by PCR, whereas 190 (66.0%) so qualified by WGS. At the individual strain level, PCR and WGS assessed ExPEC_JJ_ status concordantly for 269 strains (95.1%; 178 ExPEC_JJ_ strains and 91 non-ExPEC_JJ_ strains). Of the 19 strains with a PCR-WGS discrepancy, seven qualified as ExPEC_JJ_ only by PCR and 12 only by WGS. The 12 “ExPEC_JJ_ by WGS only” strains contained fimbrial genes (*papA* and/or *papC* and/or *afa-dra*) that were detected by VirulenceFinder but not PCR.

### Identifying UPEC_HM_ strains, correspondence with ExPEC_JJ_ strains, and crossover pathotypes.

Based on WGS typing, 201 (69.8%) of the 288 evaluation strains qualified molecularly as UPEC_HM_ (178 ExPEC_JJ_ strains and 23 non-ExPEC_JJ_ strains), whereas 87 did not (12 ExPEC_JJ_ strains and 75 non-ExPEC_JJ_ strains) (see Table S2 sheet ExPEC-UPEC). Thus, 253 strains (87.8%) were concordantly positive or negative for both UPEC_HM_ status and ExPEC_JJ_ status.

Additionally, five strains qualified for a DEC pathotype (three as EAEC and two as AEEC). All three EAEC strains were blood isolates. Two qualified as both ExPEC_JJ_ and UPEC_HM_ and contained, respectively, *afaD*, *lpfA*, ORF3, ORF4, *aap*, *aar*, *aatA*, *agg3ACD*, *agg5A*, *aggR*, and *aaiC* (strain V32) and *afaABCD*, ORF3, ORF4, *aap*, *aar*, *aatA*, *aag3ABCD*, *agg5A*, *aggR*, and *aaiC* (strain VAEC1287). The third EAEC strain (strain H8) qualified as ExPEC_JJ_ but not UPEC_HM_ and contained *astA*, *afaD*, ORF3, ORF4, *aap*, *aar*, *aatA*, *aggR*, *aaiC*, and *aggACD*. By contrast, the two AEEC strains (PUTI288 and FVEC629), which were from urine and feces, respectively, qualified as neither ExPEC_JJ_ nor UPEC_HM_. They contained multiple AEEC-associated genes (*eae*, *espA*, *espB*, *espF*, *nleB*, *nleC*, *sepA*, and *tir*) but no ExPEC- or UPEC-associated genes (Table S2).

## DISCUSSION

This study’s objective was to enhance the ExPEC-specific virulence gene database of the established web tool VirulenceFinder (9), thereby allowing enhanced *in silico* virulence genotyping of E. coli strains. The use of WGS for routine typing has already proven its value for characterization of bacterial isolates. Increasingly user-friendly tools are being developed that enable clinical health personnel without bioinformatics skills to quickly extract and interpret the relevant information from the massive amounts of sequence data (7, 9, 22, 23). Many of these tools rely on the development of curated databases to enable extraction of relevant WGS data for identification and typing purposes. Here, we built a FASTA database containing 38 putative extraintestinal virulence genes, including (according to one established operational molecular definition of ExPEC) relevant ExPEC-defining marker genes and operons and validated it extensively against PCR-based detection.

Our findings demonstrate that ExPEC-specific genes can be extracted in an automated fashion from WGS data and that the results are largely comparable to PCR results. Full (100%) concordance between PCR and WGS results was found for six of eight control strains. As for the discrepancies, only 1 gene (*ompT*) was found solely by PCR, whereas an additional 16 virulence genes (*papA*, *afaA*, *afaD*, *afaE*, *cea*, *cia*, *clbB*, *etsC*, *hlyF*, *irp2*, *iucC*, *kpsE*, *neuC*, *sitA*, *tcpC*, and *terC*), 4 fimbrial adherence genes (f17A, f17G, *fanA* [F5], *fim41a*), and 5 serotype-specific P fimbrial genes (*fsiA*-F16, *fteA*-F10, *papA*-F13, *papA*-F14, and *papA*-F536) were found only by WGS in eight of the control strains. Finally, one of the control strains (2H16) and five of the evaluation strains were newly classified as DEC.

In the 288 evaluation strains, 269 (93.4%) exhibited concordance for ExPEC_JJ_/non-ExPEC_JJ_ status. Most discrepancies involved strains that qualified as ExPEC_JJ_ only by WGS, although in 58% of these strains, the PCR primer sequences were present despite the negative PCR results. The remaining 42% of discrepancies could be explained by a lack of sequence homology between the PCR primers and the actual gene sequence.

Overall, WGS identified more target genes than did PCR. This was expected because some of the gene variants identified here were not complementary to the PCR primer sequences that conventionally have been used to identify these genes (6). For other genes (e.g., *cvaC* and *papA*), the primer pair was designed so that only one primer was located within the gene sequence *per se* (the other was outside the gene), whereas for *cia-cib*, both PCR primer-binding sites were located outside the gene. However, the “missing” PCR primer sites in the gene could not explain all instances in which PCR-negative strains were WGS positive or any of the PCR-WGS discrepancies for the 152 evaluation strains in which the target gene in question contained both primer sequences. Repeat PCR testing resolved most of these discrepancies for seven of the strains, which implicates experimental error or clerical error as the basis for the initial discrepancies. Other possible explanations for PCR-WGS discrepancies include strain substitutions and nonselection of the same colony for both WGS and PCR. Such discrepancies have been amply documented in other contexts (24). Poor sequence quality or uploading of wrong data could also explain some of the disagreements between PCR and WGS.

We also identified new *papA* alleles that contained the consensus *papA* forward primer sequence but for which no allele-specific reverse primer had been designed because they were from studies with a different purpose (25) or were submitted directly to NCBI (GenBank accession number CP019944 from a chicken carcass). By contrast, with inference of a strain's capsular K type from its putatively serotype-specific *kpsM* alleles, which we do not recommend, we do believe that the *papA* alleles, which have been shown to encode the serotype-specific part of P fimbriae (15), can be used directly via the revised VirulenceFinder to identify (F) serotype-specific PapA variants. By combining the updated VirulenceFinder with the SerotypeFinder (7), it is, therefore, possible now to serotype E. coli isolates *in silico* for O:H and F (P fimbriae) antigens based on WGS data.

When downloading genes for the ExPEC database, we noted multiple incorrectly assigned genes. These included 3 *ompT* alleles that actually encode a GlcNAc transferase instead of an outer membrane protease and 37 *focC* alleles that by BLASTx actually represent *fimC*. Notably, *focC* and *fimC* exhibit 68% DNA sequence identity, and their gene products are both chaperone proteins, specific for the respective fimbrial types (i.e., F1C fimbriae and type 1 fimbriae) (26). Likewise, four of the downloaded *sitA* gene alleles were from non-E. coli genera (*Klebsiella* and *Citrobacter*) (see Text S1 in the supplemental material). Except for the noninclusion of incorrectly assigned genes and the change of *draD* to *afaD* in the database, no further actions were taken.

The reverse problem with incorrect gene name assignments was that our initial name-based NCBI search failed to identify some gene variants because they were assigned under a different gene name. This was the case for *papA*, encoding the major F antigen-specific fimbrial adhesin PapA, for which different (antigen-specific) gene names were found, including *feiA* (F8), *fteA* (F10), *ffiA* (F15), and *fsiA* (F16). Similarly, one allele of the three F14-specific variants was named *ffoA*, whereas the F7-2-specific allele was nameless but was similar to the *pixA* genes, which were not included in the database. Finally, one allele, with 99.1 to 99.7% identity to seven F11-specific alleles, was designated F1651A (GenBank accession number ECOF165A).

A special gene name challenge involved the afimbrial *afa-dra-daa* and aggregative *aggB* genes and the *sfaD-focI* and *sfaE/focC* allele pairs, for which different names were sometimes used for sequences that were identical or nearly so. Revision of the nomenclature might be necessary, possibly involving those authors who initially created and/or assigned the abovementioned gene names. However, such a revision exceeded the scope of this study. These findings all confirm the importance of validating the genes in databases used for typing of bacterial strains and the importance of a standardized nomenclature.

Our searches for alleles used a default identity criterion of 60%. Although this conceivably could have excluded alleles with functions identical or similar to the target genes, it was adopted so as to minimize irrelevant variants and to maximize database validity. However, for one gene variant, *yfcV*, a more stringent threshold was needed to ensure database validity.

Users of this database should interpret the results prudently, remembering that genotype does not reliably predict phenotype. Many bacterial strains, both encapsulated and acapsular, contain homologues to various E. coli group II capsule genes. Therefore, special caution is advised with detection of *kpsM* alleles, which do not reliably indicate that a capsule is expressed or that a strain necessarily represents ExPEC if it contains only one additional ExPEC_JJ_-defining gene. This was shown for E. coli BL21 (DE3), a descendant of the nonpathogenic E. coli strain B, which contains a chromosomal gene cluster characteristic of group II-encapsulated strains but does not express a capsule (27). The same applies for the *kpsM* III allele, which should not be regarded as indicating that the source strain necessarily expresses a group 3 capsule. This is illustrated by control strain J96, which is positive for *kpsM* III by WGS but according to serological testing is acapsular (28).

Conversely, PCR identified *hra* more often than did WGS. In the evaluation set isolates, PCR detected *hra* in 60% of the blood and urine isolates but in only 26% of the fecal isolates, a very similar result to the overall finding of *hra* in 55% of 486 UTI strains compared to 28% of 165 rectal strains (*P = *0.001) by Srinivasan et al. (29). By contrast, WGS detected *hra* in only 33% of the blood and urine isolates, similar to the 29% observed for fecal isolates. These results suggest that PCR may detect truncated *hra* genes in non-ExPEC strains. Truncated genes with 100% identity to *hra* in reference strain PCN033 were noted in non-ExPEC strains ATCC 43888 (a non-Stx-producing O157:H7 reference strain [GenBank accession number CP041623]) and CFSAN067215 (an O18:H1 food isolate [GenBank accession number CP028320]). In both instances, the *hra* primers matched 100%.

The low similarity (∼60 to 75%) found among the available putative *hra* alleles raises concern regarding the confidence in the uniformity of this gene as *hra*. The original *hra* gene (*hra1*) (21) is a 90% identical allelic variant of *hek*, reported from uropathogenic E. coli and neonatal meningitic E. coli (29, 30). It shares 67% identity with the outer membrane invasin and adhesin Tia (31, 32). The *tia* gene has been reported as widely disseminated, but many of the strains initially thought to carry *tia*, including the genome-sequenced EAEC strain 042 (GenBank accession number NC_017626.1), actually have *hra1* (32, 33). Thus, the association of *hra* and ExPEC awaits more detailed examination.

Our addition of new ExPEC alleles to the existing VirulenceFinder web tool allowed the novel identification of multiple genes in the study isolates (9 control strains, 288 evaluation isolates), thereby providing a more complete picture of their genetic makeup. This is exemplified by our finding that five evaluation set isolates also represent DEC, including three EAEC blood isolates that were crossover pathotypes (ExPEC-EAEC) and one AEEC urine isolate and one AEEC fecal isolate that were classified as non-ExPEC/non-UPEC_HM_. The original VirulenceFinder would have identified the genes characterizing the DEC isolates but would not have classified the three crossover ExPEC-EAEC isolates as such.

Even though the E. coli VirulenceFinder database existed before this study, the present addition of curated gene alleles for 38 ExPEC-associated genes and alleles thereof should allow VirulenceFinder users to more completely characterize WGS data from E. coli isolates. The revised database includes the genes used in established molecular definitions of ExPEC and UPEC (4, 5) and will facilitate future studies of the relevance of these and other definitions. This has become increasingly important, considering the plasticity of the E. coli genome and the increasing number of reports of so-called hybrid or crossover pathotypes. Such strains contain the defining genes for both extraintestinal and intestinal pathotypes or multiple intestinal pathotypes. An example is the Stx-producing EAEC O104:H4 strain that caused the largest known STEC outbreak in Germany in 2011; that strain apparently evolved by a relatively harmless EAEC strain acquiring the Stx2a-producing bacteriophage (34). Another example is the O78:H10 clonal group (ST10; phylogenetic group A) that caused an outbreak of urinary tract infections in 1991 in Copenhagen, Denmark; that lineage exhibited characteristics of both ExPEC and EAEC (35). Most recently, the extended VirulenceFinder was also used to demonstrate the emergence of new crossover ExPEC-EAEC variants within the Escherichia coli ST131 *fimH27* subclone that harbor AggR and AAF/V fimbriae and caused bacteremia in Mozambican children (36).

In conclusion, this study shows that VirulenceFinder is able to extract ExPEC-specific genes from uploaded WGS data in a reliable and user-friendly manner, which makes this important function accessible to non-bioinformatics users worldwide. Our validation analysis demonstrated that *in silico* typing using WGS data yields results that in several respects are more detailed and complete than those obtained by established multiplex PCR methods. However, users of the database must be cautious when interpreting the results and, to avoid incorrect gene identification, should always consider the thresholds used. Important limitations of the tool are that it cannot be used to predict phenotype nor does it assign specific pathotypes; users can apply whatever algorithms they like (whether manually or automatically) to the data provided by VirulenceFinder, thereby classifying their genomes according to any classification scheme that is based on such data. Finally, we encourage users to contact us with suggestions for relevant ExPEC-associated virulence genes for possible addition to the VirulenceFinder database.

## Supplementary Material

Supplemental file 1

Supplemental file 2
