# Supplementary material for: In Silico Genotyping of Escherichia coli Isolates for Extraintestinal Virulence Genes by Use of Whole-Genome Sequencing Data
Source: J Clin Microbiol. 2020 Sep 22;58(10):e01269-20. doi: 10.1128/JCM.01269-20 (PMC7512150; doi:10.1128/JCM.01269-20)
Supplement: Supplemental file 2 [file JCM.01269-20-s0002.pdf]

Supplementary material 1

Supplementary material and methods:

Whole-Genome Sequencing

Pure bacterial cultures were cultured overnight at 37°C on SSI 5% blood agar plates (SSI Diagnostica, Hillerød, Denmark). Genomic DNA was purified with Qiagen DNeasy Blood and Tissue Kit (Qiagen, Valencia, USA) according to the kit protocol. Initial DNA concentration was measured and quantified using the Qubit Fluorometer and dsDNA BR/HR Assay Kit (Thermo Fisher Scientific). Sample and library preparation was performed using the Nextera XT v2 DNA Library Preparation kit. Libraries were finally purified by Agencourt AMPpure XP System (Beckman Coulter, Indianapolis, USA), and whole-genomes were sequenced using an Illumina Nextseq with paired-end technology (250 base pair reads). Reads were assembled using the DeBruijn graph-based de-novo assembler SKESA v.2.2 designed for assembling reads using Illumina.

(Average read coverage at 22 was set as a preliminary quality assessment.)

Sequences are available at the European Nucleotide Archive (https://www.ebi.ac.uk/ena/data/view/PRJEB38689) with accession numbers ERS4600802 (strain 31A) and ERS4600803 (strain 11A).

Sequence Analysis

Sequence reads were

1) *de novo* assembled using CLC Genomic Workbench (Qiagen) with default settings and a minimum contig size of 500 bp. Statistics from the genome assembly were used as further quality filtering and N50 values higher than 50.000 were accepted.

2) blasted against our prototype *VirulenceFinder* database using the CGE web tool MyDb,

Sequence types (ST) based on the seven gene MLST scheme for *E. coli* were determined from the de novo assembled genomes using the web tool MLST 1.8 (https://cge.cbs.dtu.dk//services/MLST).

ExPEC genes identified by VirulenceFinder in validation strains:

ExPEC genes not identified by VirulenceFinder in validation strain L31:

For the control strain, L31, 6 of the genes and one operon found by PCR could not be identified by WGS (*clbB, hra, kpsM, papC, sfaS* and *sfa/focDE*). The primer sequences for these genes along with PCR primers for *pic*, allele I' and allele I (2) could not be identified either by MyDbFinder. The strain was therefore removed from the further analysis, as we were unsure if the correct sequence data for the strain had been uploaded to NCBI.

Supplementary Results

Exclusion of gene variants from the database:

One *cvaC* allele (JHDL0100011) was removed as it was <61% identical to the other *cvaC* alleles.

Three *ompT* alleles (CYBK01000048, UDAJ01000003 and DQ381420) were removed as they were found to be GlcNAc transferase by Blastx.

Eleven *pap*A alleles were 48-67% similar to the 41 *papA* alleles but were removed from the database as BLASTx found them to resemble *pixA* (Acc. No. AJ307043) beta-globin found in *Salmo salar* (Acc. No. Y08923), or other fimbria than the major pilus subunit *papA* (CYEJ01000020, JNNA01000073, JORH01000015, APZJ01000189, JNQX01000023, CYGN01000076, AIFJ01000006, APZX01000402, JORQ01000089).

Four *sitA* alleles were 64-73% similar to the 56 unique *sitA* alleles, but were removed from the database as BLASTx confirmed them to originate from *Klebsiella* (FLWH01000008 and FLXF01000002) and *Citrobacter* (UFZE01000001 and UGBZ01000001).

Sfa/focDE

Eighty unique putative *focC* alleles were downloaded from NCBI. Of these, only four contained the *sfa/focDE* reverse primer, in each instance with > 99% identity. Of the remaining 76 alleles, 39 shared < 60% identity with the four *focC* alleles, and 37 proved to be *fimC* by blastx analysis against the non-redundant protein sequence database (nr). Therefore, these 76 alleles were not included in the final ExPEC database (see Supplementary material 1 for details of the analysis). The reverse *sfa/focDE* primer was located in all three unique *sfaE* alleles, with > 98% identity. The *sfaE* and *focC* gene alleles

had identities > 98%, and two *sfaE* alleles and two *focC* alleles were 100% identical. A total of 10 unique *sfaD* alleles and one *focI* allele were downloaded from NCBI. The forward *sfa/focDE* primer was found in the *focI* allele and in the 10 *sfaD* alleles. The *sfaD* and *focI* alleles were > 98% identical. In summary, two *focC*, two *focI*, 10 *sfaD*, one *sfaE*, and two *focC/sfaE* alleles were added to the ExPEC database.

The original VirulenceFinder database consisted of one *sfaS* allele. One additional unique allele was identified for *sfaS* (similarity of 99.8 %) and two alleles for *focG* (similarity 99.8%). The *sfaS* primer pair was found in both *sfaS* alleles, and the *focG* primer pair was found in both *focG* alleles. The *focG* forward primer and the *sfaS* forward primer were also found in both *sfaS* alleles and both *focG* alleles, respectively. The three new alleles were added to the ExPEC database. The *focG* and *sfaS* genes are ~65 % identical.

afa/dra/daa and *aggB*

Four unique *afaA* alleles were found. These had designations as *afaA*-3/*draA*-3, *daaA*, *afaA*-1, and *afaA*-8 at NCBI, but are called *afaA* in the ExPEC database. Six unique *afaB* alleles were found, including five *afaB*-1 alleles (> 99% identical) and one *afaB*-8 allele (69-71% identical to the five *afaB*-1 alleles). However, three of the *afaB* alleles were 100% identical to three *nfaE* alleles already present in the *E. coli* VirulenceFinder database. This resulted in the addition of only 3 *afaB* alleles to the ExPEC database. Eleven *afaC* alleles were added to the ExPEC database, including three unique alleles each for *afaC*-1, *afaC*/*draC*, and *afaC*-8, and two for *afaC*-3.

Thirty unique *afaE/draE/daa* alleles were added to the ExPEC database. These included 14 unique *afaE*-3/*draE* alleles, with ≥ 99.17 identity to one another; nine Dr adhesin (*afa/dra*) alleles, with ≥ 99.17 identity to one another and 70.81-71.43% identity to *afaE*-3/*draE*; one *daaE* allele (F1845), with 63.33-64.17% identity to the Dr adhesins; and one *afaE*-1, one *afaE*-2, one *afaE*-5, and three *afaE*-8 alleles, with ≥ 98.25% identity to one another.

afaD

Thirty-seven unique *afaD* alleles were found at NCBI being >36% identical. Thirteen alleles (similarity > 98%) clustered together and were confirmed to be *afaD* by BLASTx. Three *afaD*-3 alleles (JOST01000124, JNQU01000050, and JNPU01000066) were 62-63% identical with the 13 alleles described above. Two alleles were >99.8% and one allele (JOST01000124) was 100 % identical to the *agg3B* allele already found in the VirulenceFinder database and therefore not included. Five alleles (UGAK01000003, UGFM01000001, UGEQ01000001, UDBH01000052 and UINT01000044.1) were

found to be either aggregative adherence fimbria 3 minor subunit Agg3B or *afaD* by BLASTx. They were submitted using different names for very similar genes e.g., EAEC strain "NCTC7927" was submitted as both *afaD* and "Protein Agg3B, putative invasin" (acc. No. UGAK01000003). Eight *afaD*-2 alleles were 93-94% identical to the 13 alleles confirmed to be *afaD*, and were found to be adhesins or hypothetical proteins by BLASTx. One allele was from *afaD*-7 (AF072901), two were from *afaD*-8 (GQ916596 and AF072900), and two unknown (UGDD01000003 and LT985252) and all had similarities between 43-60% to all other *afaD* alleles. One allele (UGDH01000002) was 57-59% identical to the 13 alleles, and was found to be aggregative adherence fimbria 4 minor subunit Agg4/HdaB by BLASTx and was 99.5 –99.8 % identical to two *agg4B* alleles already in the *VirulenceFinder* database. Another three *agg4B* alleles (UGBU01000001, UGCQ01000001, UGCK01000004) > 98.2% to *agg1B* and >97.7% to *agg2B* already in the *VirulenceFinder* database. Thus, a total of 37 unique *afaD* alleles were added to the ExPEC database, including alleles classified as either Agg3B, Agg4/HdaB and/or *afaD*. All *draD* labels were changed to *afaD*.

kpsM:

When searching for the *kpsM* alleles, preference was given to sequences where information on the serotype included the K capsule K antigen and/or the original strain number was provided. Eighty-two unique alleles were found for the *kpsM*. Twelve sequences were excluded because of a similarity lower than 74.3% to any other *kpsM* sequence and because there was no match to any of the *kpsM* primers. Sixty-eight alleles with a similarity higher than 90.5% were found in sequences related to group 2 capsules and two unique alleles with similarity 65.4-68.5% to the 68 group 2 alleles were found in strains indicating presence of group 2 capsules K94 and K97. The similarity between these two was 77.9%. Nine group 3 capsule alleles were found with a similarity higher than 99.5% in five sequences but with similarities of only 70.9% and 78.5% for indicated K19/K23 and K11 capsules to the five other group 3 alleles, and similarities of 46.3-48.9% for two sequences indicating group 3 capsule K19. Three alleles for *kpsM*-K15 with similarities higher than 99.7% were included. For the *kpsM* genes, seven, six, five, and three alleles matched 100% for primer pairs kii-kpsII-F/K1-kii-R, kii-kpsII-F/kii-R, kpsIII-F/kpsIII-R, and kpsM15-F/R respectively. Twenty-four, 7 and 16 alleles matched primer pair kii-kpsII-F/K1-kii-R 95.7%, 91.3% and 82.6-87% respectively, while 2 alleles had 59.1-63.6% similarity to kpsIII-F and 68.2-77.3% to kpsIII-R. Eight alleles had one base difference to K1-F and to K1/kii-R. Four alleles had no match to any of the primer pairs but sequence information indicated presence of group 2 capsules K94 and K97 (Acc. No.s UGBH01000002 and UGCE01000001) and group 3 capsule K19 (UGEX01000002, reference strain E47a for K19). In summary, the *kpsM* alleles cluster together in three distinct groups: Group 2 contains 68 *kpsM* alleles, group 3 seven alleles, and *kpsM*-15

three alleles. In addition to these, two alleles were unique for group 3 capsule K19 and one allele for each of the group 2 capsules K94 and K97, respectively.

cib/cia:

A total of 6 unique *cib* alleles and 52 unique *cia* alleles were downloaded from the NCBI. One *cia* allele (6) was added manually because it has not been uploaded to the NCBI database. As the primer pairs for colicins *cia* and *cib* (1) do not bind within the colicin genes, they could not be used for validation of the gene variants downloaded from the NCBI. All 6 *cib* alleles clustered together and were 98.0-99.8% identical. The *cia* alleles were 52.4-99.9 % identical. Eighteen *cia* alleles were found to be more than 90% identical to the *cib* alleles, four of these were 100% identical to one of the *cib* alleles. The *cia* and *cib* genes have been described as being very similar in the N-terminal (98% identical), whereas the C-termini are only 77% identical (6). The genes from that study were downloaded and the *cia* and *cib* genes were compared. Alleles having a similarity of less than 90 % to the genes described in the paper were removed. This resulted in 6 *cib* alleles (98.0-99.9 % similarity) and 37 *cia* alleles (58.9-99.9% similarity).

hra

Gene designation *hra* was only found once in the less pathogenic porcine ExPEC strain PCN033 using the search string described in the M&M section. The *hra* gene name was not used in the sequence for the pathogenic porcine ExPEC strain PCN061 (4), where the gene was actually found by BLASTn. Therefore, the search also included literature searches for the *hra* gene and an additional 131 alleles were found. The similarity set at 60% identified *hra* alleles belonging to non-ExPEC isolates such as the EAEC strain 60A (5), and the above mentioned PCN033 strain with similarities less than 72.5% to the ExPEC related sequences of *hra* resulting in a total of eleven *hra* alleles in the database.

Genes not covered by the primers:

Twenty-seven unique *etsC* alleles were downloaded from the NCBI, which clustered into three groups. One group contained 14 alleles (>87% similar) and the second group contained 3 alleles (> 90% identical). The third group consisted of 9 *etsC* alleles (similarity > 98%), which were deleted as they were found to be TssF by BLASTx. This resulted in 18 unique *etsC* alleles. A total of 21 unique *kpsE* variants were identified. Eighteen of them were >96% identical. The remaining three *kpsE* alleles were 49.9-52.6% identical to the 18 alleles described above. Forty-seven unique *iucC* alleles (similarity > 95%) were found. Sixty-eight unique *neuC* alleles were found being > 45% similar. The alleles cluster

together in several groups, where the internal identity is >99%. BLASTx confirms them all to be UDP-N-acetyl glucosamine 2-epimerases. Four *tcpC* gene alleles were found with a similarity of >99 %. One allele was removed as it was shorter than the other three alleles (720bp vs. 924bp) and was reported as being truncated (GQ902993). Twenty-five unique *terC* alleles were found, of them 19 were > 96% similar, three of them were > 98% similar and >80% similar to the before mentioned 19 *terC* alleles, another three alleles differed more from the first 22 alleles, and are >52% similar to them. BLASTx confirms them all to be *terC* alleles.

papA genes not covered by the search string and inclusion criteria

The search string for *papA* did not return the expected allele results for the serotype-specific P fimbriae variants F8, F10, F12, F15, and F40. Therefore, an additional BLASTn search at NCBI Genbank was performed using the corresponding reverse primers and original Accession numbers (3), which had different gene names: *feiA* (F8), *fteA* (F10), *ffiA* (F15), and *fsiA* (F16). One allele of the three F14 specific variants was named *ffoA* and the F7-2 specific allele was nameless but was similar to the *pixA* genes, which were not included in the database. Finally, one allele with 99.1-99.7% identity to seven F11-specific alleles was designated F1651A (Acc. No. ECOF165A). Two new *papA* alleles were found and given the provisional names F19 (Accession No. AP017620) and F20 (Accession No. CP019944).

The lengths of the *papA* sequences varied from to 324 bp (F20, Accession No. CP019944) to 585 bp (F7-1, Accession No. AF447814). Identities below 60% were 54.2 % between F12 (Accession No. X62157) and F19 (Accession No. AP017620), and 58.3 % between F12 (Accession No. X62157) and F20 (Accession No. CP019944). Table 1 lists the number of alleles for each of the *papA* genes.

Table 1. Number of P fimbriae related genes and their relation to the F serotype

Gene	F7-1	F7-2	F8	F9	F10	F11	F1651A	F12	F13	F14	F15	F16	F19	F20	F40	F43	F48	F536	Total
feiA			2																2
ffiA											1								1
ffoA										1									1
fsiA												2							2
fteA					1														1
papA	3	1	1	1		7	1	4	4	2			1	1	1	1	3	4	35
SUM	3	1	3	1	1	7	1	4	4	3	1	2	1	1	1	1	3	4	42

Concordance of PCR and WGS typing of the evaluation sequences.

The *sfaS* (5 times), *focG* (10 times), *fyuA* (10 times), *iutA* (21 times), *ibeA* (7 times) and the *sfa/focDE* operon (8 times) were identified by WGS in PCR negative strains in the number of strains listed in parentheses. The alleles identified by WGS in these strains were positive for both of the PCR primers.

In four strains, only one of the *sfa/focDE* operon genes were identified (*sfaD, sfaE, focI, focC*) by WGS, and the strains were classified as *sfa/focDE* negative.

Thirty-three strains were positive for *papA* by WGS, all with an allele where the forward PCR primer was identified. Twenty strains negative for *papC* by PCR were found *papC* positive by WGS. For 9 of the 20 strains, the *papC* PCR primer sequences were not identified in the *papC* gene sequence. *hlyF* (1), *usp* (7), *traT* (12) and *ompT* (19) were identified by WGS in PCR negative strains with alleles, where only one PCR primer or no PCR primers were identified in the gene sequence.

Repeat PCR was performed for 7 strains and resulted in reclassification of six strains as non-ExPEC instead of ExPEC, in agreement with VirulenceFinder. The seventh strain (PUTI_374) was found to be ExPEC by VirulenceFinder, but not by PCR in either the initial or repeat testing.

References

1. **Gordon DM, O'Brien CL.** 2006. Bacteriocin diversity and the frequency of multiple bacteriocin production in *Escherichia coli*. Microbiology 152:3239-44. doi:10.1099/mic.0.28690-0.
2. **Johnson JR, Porter S, Johnston B, Kuskowski MA, Spurbeck RR, Mobley HL, Williamson DA.** 2015. Host Characteristics and Bacterial Traits Predict Experimental Virulence for *Escherichia coli* Bloodstream Isolates From Patients With Urosepsis. Open Forum Infect Dis 2:ofv083. doi:10.1093/ofid/ofv083 [doi];ofv083 [pii].
3. **Johnson JR, Stell AL, Scheutz F, O'Bryan TT, Russo TA, Carlino UB, Fasching C, Kavle J, Van DL, Gaastra W.** 2000. Analysis of the F antigen-specific papA alleles of extraintestinal pathogenic *Escherichia coli* using a novel multiplex PCR-based assay. Infect Immun 68:1587-1599. doi:https://doi.org/10.1128/iai.68.3.1587-1599.2000.
4. **Liu C, Zheng H, Yang M, Xu Z, Wang X, Wei L, Tang B, Liu F, Zhang Y, Ding Y, Tang X, Wu B, Johnson TJ, Chen H, Tan C.** 2015. Genome analysis and in vivo virulence of porcine extraintestinal pathogenic *Escherichia coli* strain PCN033. BMC Genomics 16:717. doi:10.1186/s12864-015-1890-9 [doi];10.1186/s12864-015-1890-9 [pii].
5. **Mancini J, Weckselblatt B, Chung YK, Durante JC, Andelman S, Glaubman J, Dorff JD, Bhargava S, Lijek RS, Unger KP, Okeke IN.** 2011. The heat-resistant agglutinin family includes a novel adhesin from enteroaggregative *Escherichia coli* strain 60A. J Bacteriol 193:4813-4820. doi:JB.05142-11 [pii];10.1128/JB.05142-11 [doi].
6. **Mankovich JA, Hsu CH, Konisky J.** 1986. DNA and amino acid sequence analysis of structural and immunity genes of colicins Ia and Ib. J Bacteriol 168:228-36. doi:10.1128/jb.168.1.228-236.1986.
